# Supplementary material for: Simultaneously enhancing the ultimate strength and ductility of high-entropy alloys via short-range ordering
Source: Nat Commun. 2021 Aug 16;12:4953. doi: 10.1038/s41467-021-25264-5 (PMC8368001; doi:10.1038/s41467-021-25264-5)
Supplement: Supplementary file 1 — Supplementary Information [file 41467_2021_25264_MOESM1_ESM.pdf]

Supplementary Information for

Simultaneously Enhancing the Ultimate Strength and Ductility of High-Entropy Alloys via Short-Range Ordering

Shuai Chen[1], Zachary H. Aitken[1], Subrahmanyam Pattamatta[2], Zhaoxuan Wu[2], Zhi Gen Yu[1], David J. Srolovitz[3,*], Peter K. Liaw[4,*], Yong-Wei Zhang[1,*]

[1]*Institute of High Performance Computing, A*STAR, 138632 Singapore*

[2]*Department of Materials Science and Engineering and Hong Kong Institute for Advanced Study, City University of Hong Kong, Hong Kong SAR, China*

[3]*Department of Mechanical Engineering, The University of Hong Kong, 7/F, Haking Wong Building, Pokfulam Road, Hong Kong SAR, China*

[4]*Department of Materials Science and Engineering, The University of Tennessee, Knoxville, TN 37996, USA*

[*] Email: srol@cityu.edu.hk; pliaw@utk.edu; zhangyw@ihpc.a-star.edu.sg

Table of Content

Supplementary Discussion

Supplementary Figures 1-14

Supplementary Tables 1-2

Supplementary References

Supplementary Discussion

Effect of strain rate on mechanical property of HEA

Li et al.[1] formulated a theory connecting strength (σ) and strain rate (ε) in a dislocation nucleation kinetics-based model:

$$\sigma = \frac{\Delta U}{SV^*} - \frac{k_B T}{SV^*} \ln \frac{d\nu_D}{\lambda\varepsilon} \tag{1}$$

where ΔU is the activation energy, S is a factor representing the local stress concentration and geometry, V^* is the activation volume, $k_B T$ is the thermal energy, ν_D is the Debye frequency, d and λ are microstructure parameters. Following their approach, we simplify this relation as:

$$\sigma = C + K \ln \varepsilon \tag{2}$$

where C and K are constants, which we determine from the simulation ultimate stresses (of the 0 and 4M samples) at 2×10^8 s^{-1} and 2×10^7 s^{-1} strain rates. Using Eq. (2) and the values of C and K, we estimate the ultimate stresses of these two samples for a strain rate of 2×10^{-3} s^{-1}; i.e., the ultimate stress for the 4M and 0 samples to be 3.92 GPa and 3.13 GPa, respectively. These results shows that the sample with SRO has a higher ultimate stress than the sample without SRO even at a low strain rate. Therefore, our simulation results at ultra-high strain rates suggests a similar trend in mechanical properties at low strain rates.

We note that Zhao et al.[2] observed the formation of amorphous phases in a CoCrFeNiMn HEA under a strain rate of 6×10^5 s^{-1}. These amorphous phases were attributed to the high defect density from the initial pre-processing conditions[2]. However, the initial structure of our CoCuFeNiPd HEA is perfect (i.e., contains no defects). As a result, the nucleation of amorphous phases is suppressed, even at high strain rates of 2×10^8 and 2×10^7 s^{-1}.

Optimal FCCP/BCCP fraction in HEAs

We performed additional simulations with more iterations, starting from the 4M sample to further enhance the SRO. We calculated the sum of squares of the WCPs for each MC attempt and only retained the changes that increased the sum of squares. After 10^4 successful MC iterations, we obtain sample 4M+ which has a similar potential energy (Supplementary Figure 5a) but larger FCCP and BCCP fractions (Supplementary Figure 5b). The FCCP and BCCP fractions are 33% and 9% in the 4M+ sample, 32% and 8% in the 4M sample, and 31% and 7% in the 2M sample. Therefore, the differences in the FCCP and BCCP fractions between the 4M+ and 4M samples are as much as those between the 4M and 2M samples. In our previous calculations (from 0 to 4M samples), the acceptance of each swap conformed to the Metropolis criterion, i.e., the swap with probability higher than / equal to randomly-generated number was also accepted. However, from 4M to 4M+ samples, each swap was only accepted when the sum of squares of the WCPs was increased, leading to the distinct difference after 10^4 successful MC iterations. The WCPs of 4M+ sample (Supplementary Figure 5c) show a higher degree of SRO than for the 4M sample (Fig. 1g). The tensile stress-strain curves for the 0, 2M, 4M, and 4M+ samples (Supplementary Figure 5d) shows that the largest ultimate tensile strength is obtained for the 4M sample. Detailed analysis of the 4M+ atomic configurations at 9% and 10% strains (Supplementary Figure 5e) shows that the deformation mechanism of the 4M+ sample is the same as that of other samples. Therefore, these simulation results demonstrate that there are optimal fractions for FCCP and BCCP structures. Our results demonstrate that SRO increases the BCCP fraction and makes the BCCP domain easier to transform to BCC phase. This is why the SRO alloys (the 2M, 4M, and 4M+ samples) have better mechanical properties than the random alloy. Further increase in the BCCP fraction leads to more BCC phase formed, inducing more phase boundaries in the 4M+ sample than in the 4M sample. Since dislocation nucleation occurs at the FCC/BCC phase boundaries (Supplementary Figure 2),

this implies more/easier dislocation nucleation in the 4M+ sample than in the 4M sample. This explains why there is an optimal BCCP fraction structure.

The FCC to BCC transformation occurs in BCCP clusters, which are enriched in Fe and Pd. Therefore, the key to adjusting SRO and related phase transformation in the CoCuFeNiPd HEA is to tune the BCCP cluster Fe and Pd concentrations. The change in the local concentration of Ni may originate from FCCP clusters (primarily Co and Ni) or IND clusters (consisting of all five elements). If the change in the local concentration of Ni occurs in the FCCP clusters, it may have a minor effect on the BCCP clusters and phase transformation. However, if the change comes from the IND clusters, it may introduce more BCCP clusters. Therefore, it may also affect the related phase transformation.

The variations of phase transformation and stress as a function of strain for the 0 and 4M samples for a 300 K tensile test are shown in Supplementary Figure 6a. These results indicate that the critical strain for phase transformation in the 4M and 0 samples are ~2.6% (continuous blue line) and 4% (dashed blue line), corresponding to critical stresses of 1.6 GPa (continuous red line) and 2.3 GPa (dashed red line), respectively. Atomic configurations of the 0 and 4M samples at the critical strain and stress for phase transformation are shown in Supplementary Figures 6b and c. The sample with greater SRO (4M) has lower critical stress for phase transformation due to the formation of more BCCP clusters.

Effect of grain boundary on mechanical property of HEA

To further study the effect of SRO on the mechanical properties of this HEA with a grain boundary, a model of two grains in the same size but with different orientations was constructed by aligning them along the [010] direction (Supplementary Figure 8c), where one grain is identical to the

previous single-crystal one (i.e., a 5-nm cube with [100], [010], and [001] directions, as shown in Supplementary Figure 8a), and the other grain is cut from the previous single-crystal one (i.e., a 5-nm cube with $\left[-\frac{1}{2}\,0\,\frac{1}{2}\right]$, [010], and $\left[\frac{1}{2}\,0\,\frac{1}{2}\right]$ directions, as presented in Supplementary Figure 8b). Following this procedure, samples consisting of two grains without SRO (the 0 sample) and with SRO (the 4M sample) can be constructed. To introduce SRO at grain boundaries between two 4M samples, 10^5 MC/MD iterations were performed in the shaded regions marked in Supplementary Figure 8c (1 nm thickness) to reduce the system potential energy. The models without SRO and with SRO are shown in Supplementary Figure 8d (0-0 sample) and Supplementary Figure 8e (4M-4M sample), respectively.

Variations of phase transformation and stress with strain for these two samples during tension at 300 K (along [010] direction) are plotted in Supplementary Figure 9a. It is seen that the 4M-4M sample (continuous red line) has a higher ultimate tensile stress than the 0-0 sample (dashed red line), indicating that SRO still improves the mechanical properties of this HEA with grain boundaries. The variation of phase transformation with strain plotted in Supplementary Figure 9a shows that the critical strain for phase transformation in both the 4M-4M (continuous blue line) and 0-0 samples (dashed blue line) is 1.8%, corresponding to the critical stress of 1.3 GPa (continuous and dashed red lines). These critical strain and stress (1.8% and 1.3 GPa) are lower than those of single-crystal samples (4M sample: 2.6% and 1.6 GPa; 0 sample: 4% and 2.3 GPa in Supplementary Figure 6a). Atomic configurations of the 0-0 and 4M-4M samples at a 1.8% strain shown in Supplementary Figures 9b and c, respectively, demonstrate that the phase transformation nucleates at the grain boundaries. Therefore, the grain boundaries lower the critical stress required for the nucleation of phase transformation. The phase transformation in the 0-0 sample propagates

toward the grain interior and along the grain boundaries with increasing the strain (as shown in Supplementary Figures 9b and c). However, the propagation of phase transformation toward the grain interior and along the grain boundaries in the 4M-4M sample is prohibited by highly-stable FCCP clusters formed in the grain interior and SROs in the grain boundaries (as exhibited in Supplementary Figures 9d and e). Besides, the SROs formed in the grain boundaries of 4M-4M sample increase the critical stress required for dislocation nucleation (4M-4M sample: 2.6 GPa, 0-0 sample: 2.2 GPa), leading to the enhancement of the ultimate strength.

Effect of annealing temperature on SRO of HEAs

To investigate the effect of annealing temperature on segregation and SRO, we performed MC and MD simulations on the CoCuFeNiPd HEA at 1,200 K. Not surprisingly, the same trends of SRO observed at 300 K (Fig. 1e to g) are also seen at 1,200 K (Supplementary Figure 10a and b). However, the stronger entropic effects at higher temperature greatly reduce enthalpic (bond energy) effects. As a result, the SRO at the higher temperature is reduced. This is consistent with the observation (Supplementary Figure 10c) that the energy per atom decreases less at 1,200 K, as compared with that at 300 K.

The tensile stress-strain curves for the HEAs with 0, 2×10^6, and 4×10^6 iterations at 1,200 K (0, 1,200K-2M, and 1,200K-4M samples) under tension at 300 K and 1,200 K (a strain rate of 2×10^8 s^{-1}) are shown in Supplementary Figure 11a. These data indicate that the ultimate stress and associated ultimate strain for the 1,200K-2M and 1,200K-4M samples (iterated at 1,200 K) are slightly lower, as compared with the 0, 2M, and 4M samples (iterated at 300 K), suggesting that the SRO in the HEAs after iteration at 1,200 K slightly reduces the strength and ductility. Examinations of the atomic configurations in Supplementary Figures 11b and c demonstrate that

the deformation mechanism remains unchanged (phase transformation from FCC to BCC/amorphous structures prior to the ultimate stress and dislocation slip afterwards).

The distributions of number fractions of atoms with $\Delta E_{FCC\text{-}BCC}$ for the 1,200K-2M, and 1,200K-4M samples are plotted in Supplementary Figure 11d, as compared with those at 300 K (the 2M and 4M samples). The curve shapes of the 1,200K-2M and 1,200K-4M samples are quite different from those for the 2M and 4M samples. There exist two plateaus at ~ - 3 × 10^{-2} eV and ~ 1 × 10^{-2} eV, respectively, for the 2M and 4M samples, indicating the formation of three categories of clusters (FCCP, IND, and BCCP clusters). However, the curve shapes of the 1,200K-2M and 1,200K-4M samples imply that there is no such cluster formation. Besides, the atomic configurations colored according to their phase stability for the 1,200K-2M and 1,200K-4M samples shown in Supplementary Figures 11e and f further confirm that their atomic configurations are different from that of the 2M sample presented in Fig. 3e, i.e., without FCCP and BCCP serving as hard and soft fillers in the matrix (IND) of the HEA to form a composite microstructure. This is why the 1,200K-2M and 1,200K-4M samples do not exhibit better mechanical properties than the 0, 2M, and 4M samples. Therefore, it is critical to control the heat treatment to get the best SRO, which then leads to the best mechanical properties via the composite-based mechanism.

Role of FCCP/BCCP in HEAs

In our HEA, both FCCP and BCCP clusters are in an FCC phase. In the 2M sample, FCCP clusters have an average composition of 28 Co, 18 Cu, 4 Fe, 48 Ni, 2 Pd (at%), while the average composition of BCCP clusters is 15 Co, 6 Cu, 31 Fe, 4 Ni, 44 Pd (at%). Two samples with the same elemental concentrations as the FCCP and BCCP clusters were constructed to perform tensile

tests. Examination of the stress-strain curves for these two samples (see Supplementary Figure 12a) verifies that FCCP is the hardening domain. Inspection of the atomic configurations of the FCCP and BCCP samples at different strains (Supplementary Figures 12b and c) demonstrates that the dominant deformation mechanism is dislocation slip in FCCP and phase transformation in BCCP. These results further confirm that BCCP clusters are metastable within the FCC phase and transform to BCC phases during tensile deformation. Such phase transformations contribute to both the plasticity and ductility of the material. Random FCC NiCo alloys with pre-existing dislocation have yield strengths dominated by solute-misfit[3]. However, NiCo has a near-zero misfit such that random NiCo alloys exhibit a very low yield strength[3]. In our simulations, the five-element FCCP clusters (29 Co, 18 Cu, 4 Fe, 48 Ni,1 Pd (at%)) exhibit SRO (obviously) not present in random NiCo alloys. Besides, FCCP clusters do not have pre-existing dislocation. Hence, it is no surprise that the FCCP clusters in the HEAs exhibit quite different mechanical properties from random NiCo alloys.

The average lattice parameters of the FCC phase for the 0, 2M, and 4M samples at a 6% strain at 300 K (no dislocations) are found to be 3.685 Å, 3.662 Å, and 3.642 Å, respectively; i.e., the average lattice parameter decreases during SRO formation. The BCC phase, on the other hand, shows average lattice parameters of 2.959 Å, 2.957 Å, and 2.965 Å for the 0, 2M, and 4M samples, respectively; i.e., only small, statistically insignificant change during SRO formation.

Validating the reliability of the atomic potential

We have checked the reliability of the atomic potential used in the study from three different aspects: 1. lattice constants, 2. cohesive energies and 3. melting points.

The lattice constant of CoCrFeNiPd HEA was measured experimentally by Ding et al.[5] and

found to be 3.67 Å. It was reported that the effect of Cu on the lattice constant of $CoCrFeNiCu_x$ (x=0, 0.2, 0.4, 0.6, 0.8, 1.0) HEAs was negligible[6] due to the similar atomic radius of Cu with Co, Cr, Fe and Ni. Hence, it is expected that the lattice constant of CoCuFeNiPd should be also around 3.67 Å. To make a comparison, we calculated the average lattice constants of the 0, 2M, and 4M samples and found that they were 3.674 Å, 3.667 Å and 3.666 Å, respectively. Clearly, these predicted values using the atomic potential match the expected value well.

We have also compared the cohesive energies of $L1_2$ AB_3 alloys for all binary combinations of {Co, Cu, Fe, Ni and Pd} calculated using the density-functional theory (DFT) and the atomic potential (Supplementary Table 2), and a good agreement has been observed. More specifically, both results indicate that the Cu-Cu pairs exhibit the highest cohesive energy, Cu_3-containing pairs (shaded in red) always have higher cohesive energies than other pairs, Co_3/Fe_3/Ni_3-containing pairs (shaded in green) always have lower cohesive energies than other pairs, and Pd_3-containing pairs (shaded in orange) are of medium energies.

Finally, we have performed additional simulations on the heating of the 0, 2M, and 4M samples from 300 K to 2300 K in a heating rate of 100 K/ns. The variations of potential energy with temperature for these three samples are shown in Supplementary Figure 14 (A dramatic increase in potential energy corresponds to the starting point of melting), which indicates the melting points are 1831 K, 1750 K and 1685 K, respectively. Experimental measurements reported that the melting points of CoCrFeNi, CoCrCuFe and CoCrCuFeNi are 1711 K[7], 1623 K[8] and 1662 K[9], and that of pure Pd is 1828 K[10]. Therefore, the melting points of the CoCuFeNiPd HEAs in this study are in a reasonable range with respect to the experimental values.

Clearly, the good agreement in the lattice constants, cohesive energies and melting points

obtained using the atomic potential and from other sources validates the reliability of the atomic potential used in the present study.

Supplementary Figures

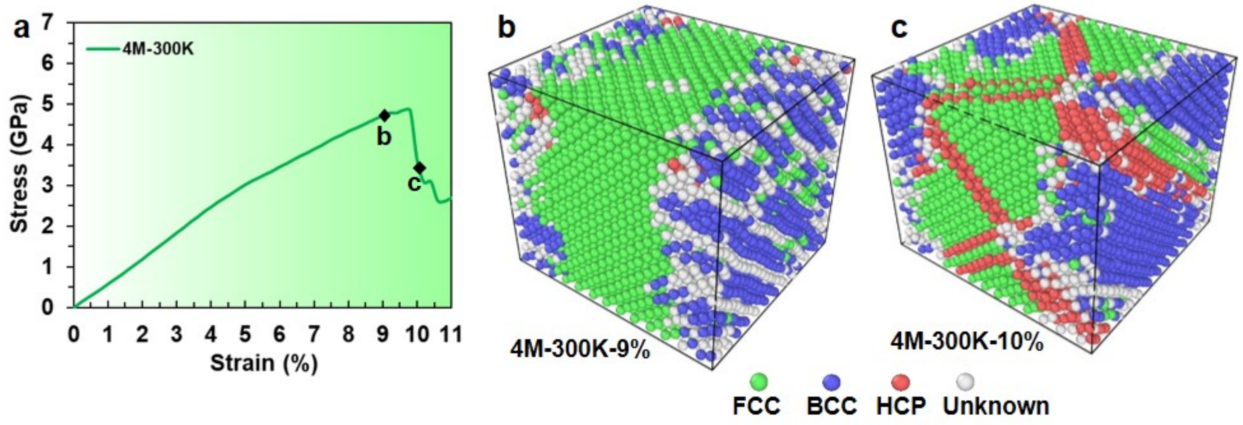

Supplementary Figure 1. Stress-strain curve and atomic configurations of the CoCuFeNiPd HEA with 4 × 10^6 iterations at 300K (the 4M sample) during tension at 300K (300K tension). a Variation of stress with strain for the 4M sample during 300K tension (4M-300K). Atomic configurations of the 4M sample at strains of **b** 9% (4M-300K-9%), and **c** 10% (4M-300K-10%) colored according to their common neighbor analysis[4] on phase structures (marked in **a**).

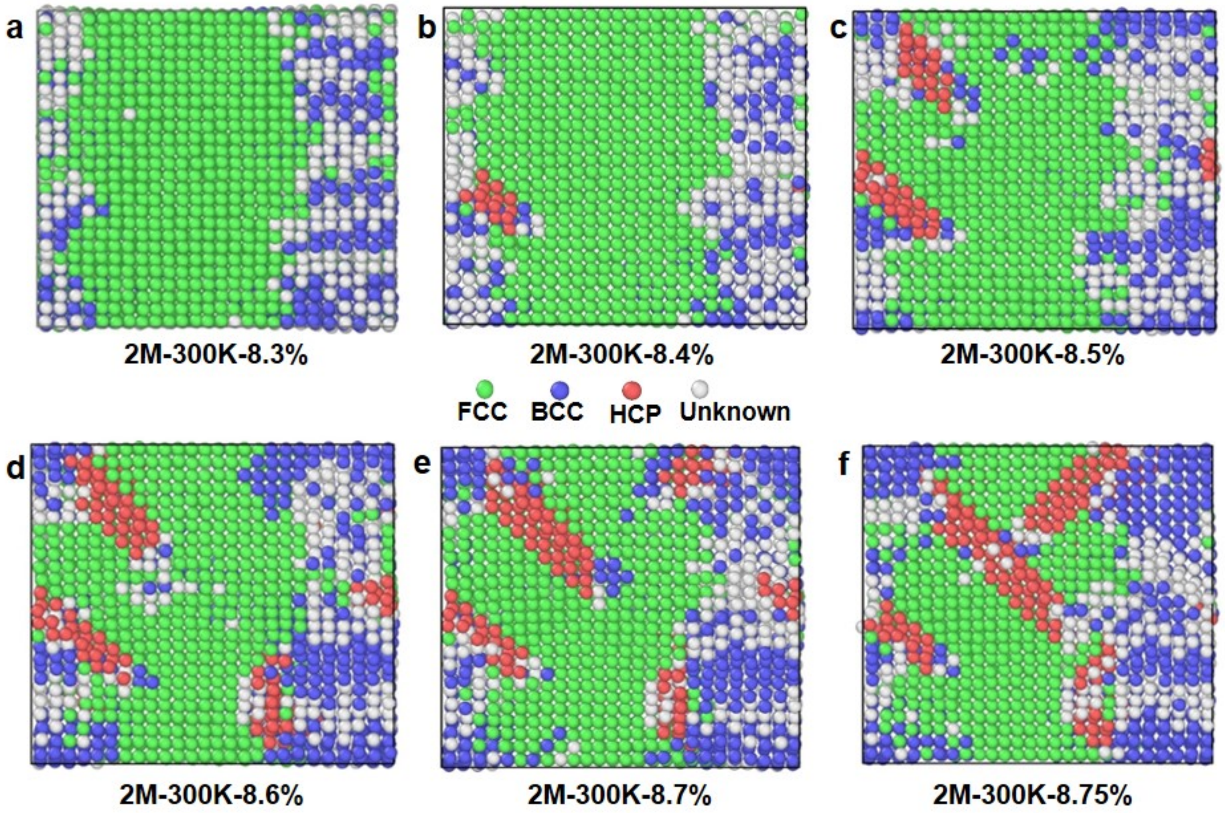

Supplementary Figure 2. Atomic configurations in a {001} cross-section of the CoCuFeNiPd HEA with 2 × 10^6 iterations at 300K (the 2M sample) during tension at 300K. Atomic configurations of the 2M sample at strains of **a** 8.3% (2M-300K-8.3%), **b** 8.4% (2M-300K-8.4%), **c** 8.5% (2M-300K-8.5%), **d** 8.6% (2M-300K-8.6%), **e** 8.7% (2M-300K-8.7%), and **f** 8.75% (2M-300K-8.75%) colored according to their common neighbor analysis[4] on phase structures.

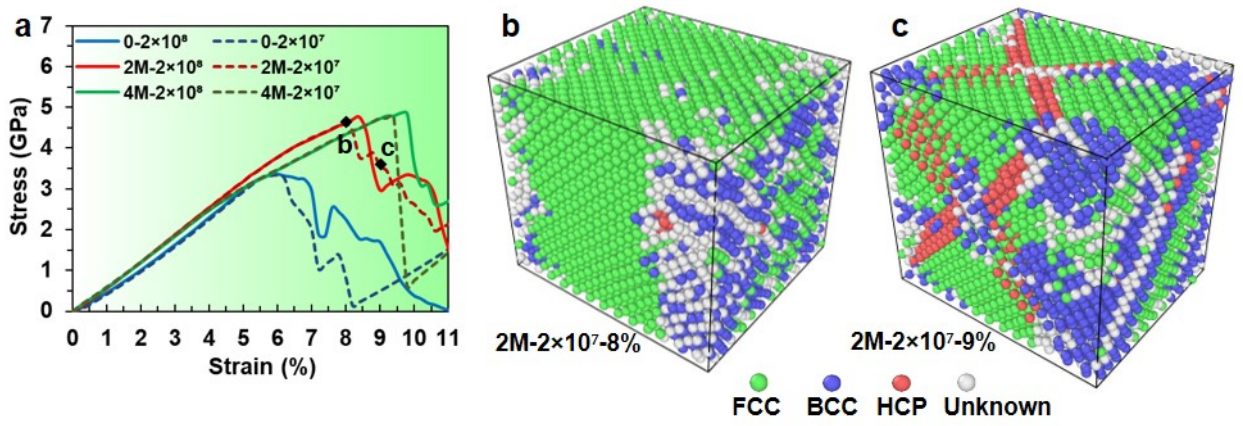

Supplementary Figure 3. Stress-strain curves and atomic configurations of the CoCuFeNiPd HEA with 0, 2 × 10^6, and 4 × 10^6 iterations at 300K (0, 2M, and 4M samples) during tension at 300K. a Variations of stresses with strains for the 0, 2M, and 4M samples at strain rates of 2 × 10^8 s^{-1} (0-2×10^8, 2M-2×10^8, and 4M-2×10^8) and 2 × 10^7 s^{-1} (0-2×10^7, 2M-2×10^7, and 4M-2×10^7). Atomic configurations of the 2M sample under a strain rate of 2 × 10^7 s^{-1} at strains of **b** 8% (2M-2×10^7-8%), and **c** 9% (2M-2×10^7-9%) colored according to their common neighbor analysis[4] on phase structures (marked in **a)**.

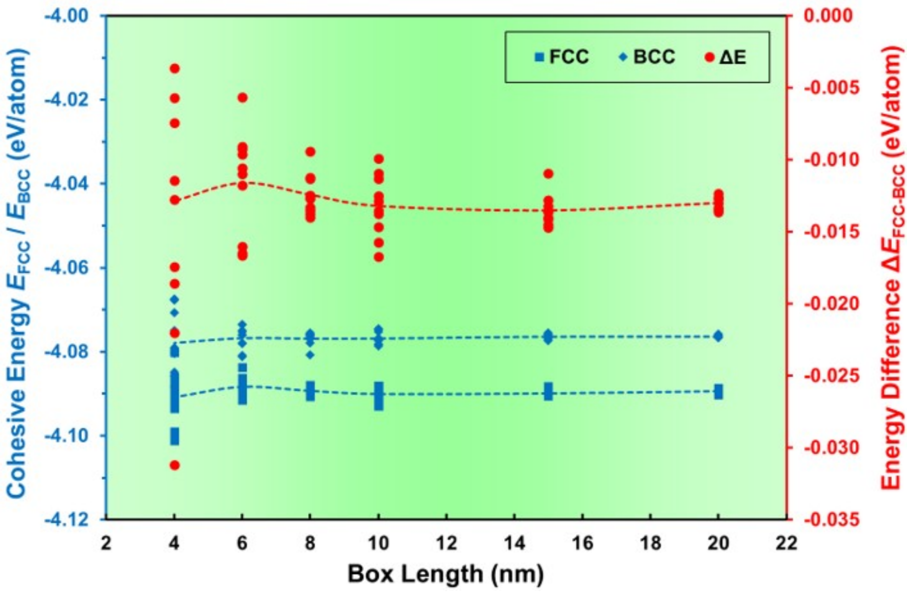

Supplementary Figure 4. Cohesive energies of FCC (E_{FCC}) and BCC (E_{BCC}) structures and their energy differences ($\Delta E_{FCC\text{-}BCC} = E_{FCC} - E_{BCC}$) with a random elemental distribution in the CoCuFeNiPd HEA as a function of the simulation-box length.

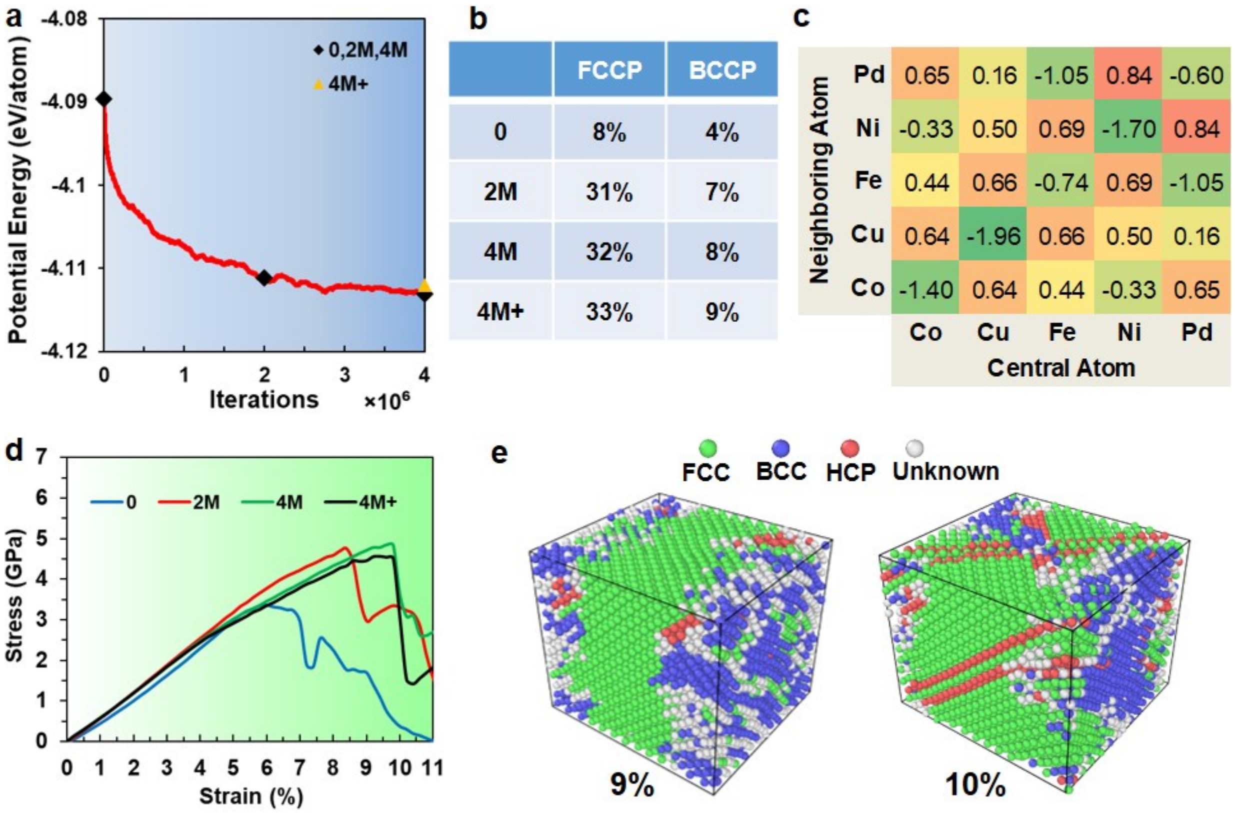

Supplementary Figure 5. Potential energies, fractions of atoms with different phase preferences, Warren-Cowley parameters, stress-strain curves, and atomic configurations of the CoCuFeNiPd HEA for different iterations at 300K. a Variation of the potential energy with iteration. **b** Fractions of atoms with different phase preferences for the HEA with 0 (0 sample), 2×10^6 (2M sample), 4×10^6 (4M sample), and $4\times10^6+1\times10^4$ (4M+ sample) iterations. **c** Warren-Cowley parameters for the 4M+ sample. **d** Uniaxial tensile stress-strain curves of the 0, 2M, 4M, and 4M+ samples during tension at 300K. **e** Atomic configurations of the 4M+ sample at 9% and 10% strains colored according to the common neighbor analysis[4] on phase structures.

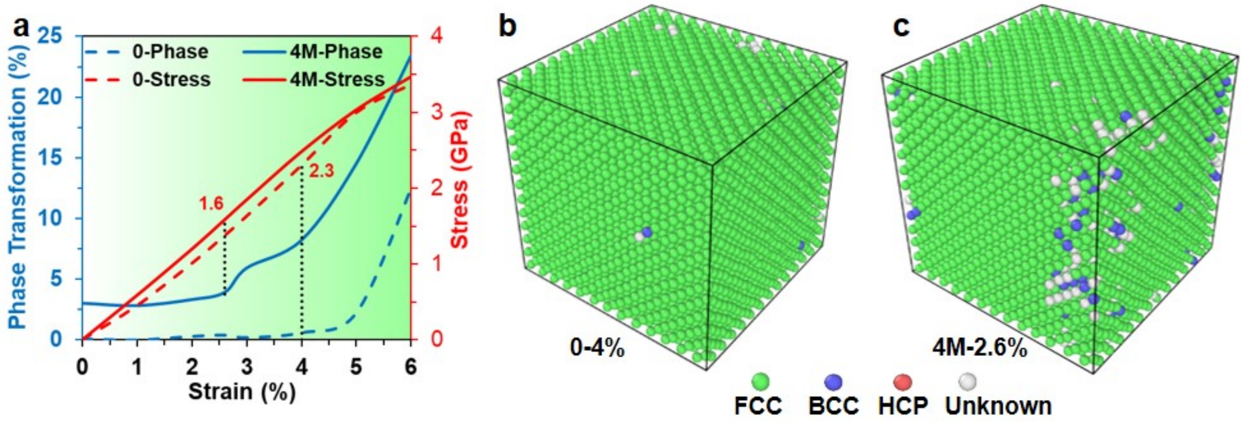

Supplementary Figure 6. Fraction of phase transformation and stress vs strain curves and atomic configurations of the CoCuFeNiPd HEA of the random (0) and 4×10^6 SRO (4M) samples tested in tension at 300 K. a Variations of phase transformation fraction and stress with strain for the 0 and 4M samples during tension at 300 K. Atomic configurations of **b** the 0 sample at a 4% strain (0-4%), and **c** the 4M sample at a 2.6% strain (4M-2.6%) colored according to their common neighbor analysis[4] on phase structures.

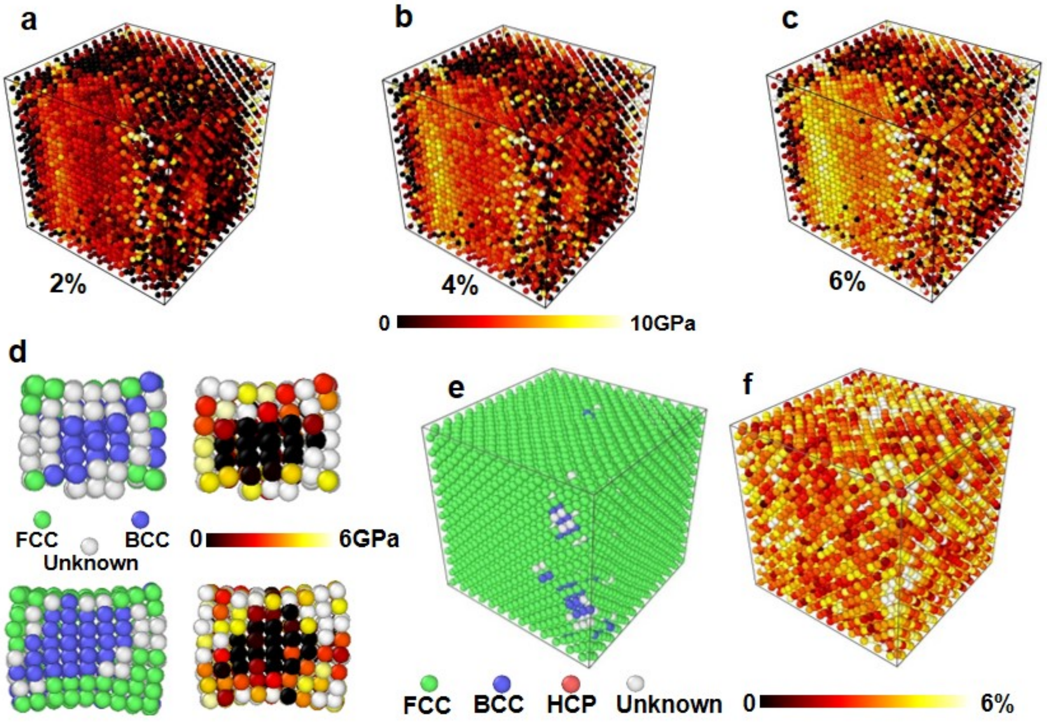

Supplementary Figure 7. Atomic configurations of the CoCuFeNiPd HEA with 2 × 10^6 iterations at 300 K (the 2M sample) during loading and unloading at 300 K (2 × 10^8 s^{-1} strain rate). Atomic configurations of the 2M sample at strains of **a** 2%, **b** 4%, and **c** 6% colored according to their stress distributions during loading. **d** Two domains in the 2M sample at strain of 8% colored according to their phase structures or stress distributions. Atomic configurations of the 2M sample colored according to their **e** phase structures, and **f** strain distributions after unloading.

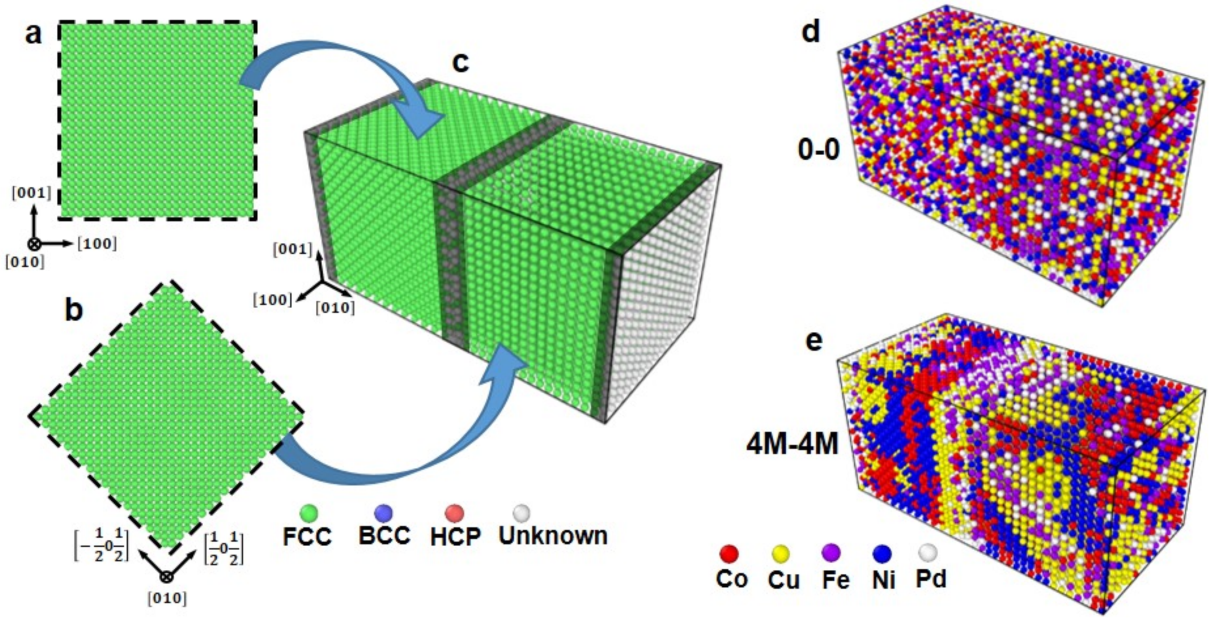

Supplementary Figure 8. Construction and atomic configurations of the CoCuFeNiPd HEAs with grain boundaries. **a** Grain 1 with orientations: [100], [010], and [001] and **b** Grain 2 with orientations: $\left[-\frac{1}{2}0\frac{1}{2}\right]$, [010], and $\left[\frac{1}{2}0\frac{1}{2}\right]$) along the [010] direction meet to form a sample **c** with grain boundaries. Atomic configurations of the CoCuFeNiPd HEAs consisting of **d** two 0 grains (the 0-0 sample**),** and **e** two 4M grains after 10^5 iterations for grain boundaries (the 4M-4M sample**)**.

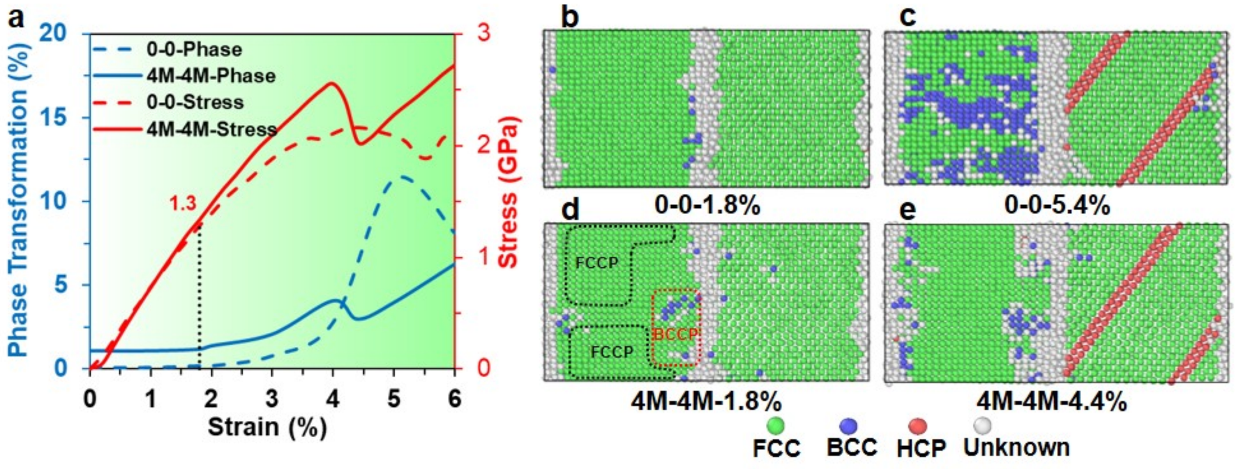

Supplementary Figure 9. Fraction of phase transformation, stress vs strain curves and atomic configurations of the CoCuFeNiPd HEAs with grain boundaries during tension at 300 K. a Variations of phase transformation fraction and stress with strain for the 0-0 and 4M-4M samples. Atomic configurations of the 0-0 sample at **b** 1.8% strain (0-0-1.8%) and **c** 5.4% strain (0-0-5.4%), and the 4M-4M sample at **d** 1.8% strain (4M-4M-1.8%) and **e** 4.4% strain (4M-4M-4.4%) colored according to their common neighbor analysis[4] on phase structures.

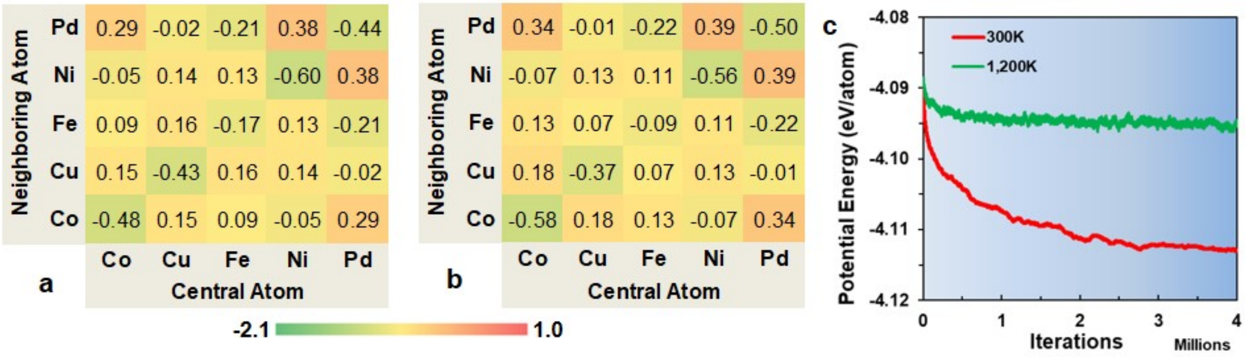

Supplementary Figure 10. Warren-Cowley parameters and potential energies of the CoCuFeNiPd HEA as a function of iterations. Warren-Cowley parameters of the HEA after **a** 2×10^6 and **b** 4×10^6 iterations at 1,200 K. **c** Variation of potential energies with iterations at 300 K and 1,200 K.

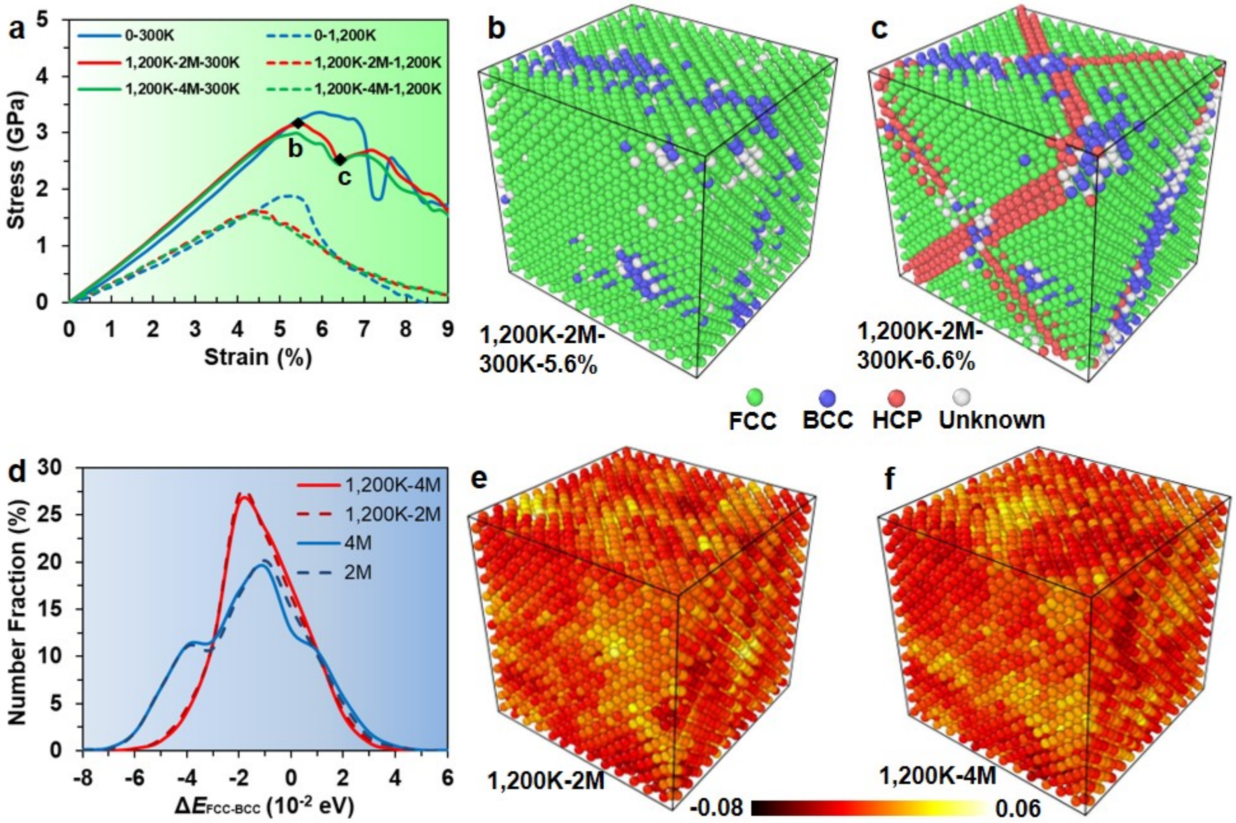

Supplementary Figure 11. Dependence of the stress-strain curves, atomic configurations, and phase stabilities of the CoCuFeNiPd HEA on number of iterations at 1,200 K. a Uniaxial tensile stress-strain curves for the HEAs with 0, 2×10^6, and 4×10^6 iterations at 1,200 K (the 0, 1,200K-2M, and 1,200K-4M samples) under tension at 300 K and 1,200 K. Atomic configurations of the 1,200K-2M sample under tension at 300 K at **b** 5.6% (1,200K-2M-300K-5.6%) and **c** 6.6% (1,200K-2M-300K-6.6%) strain, colored according to their common neighbor analysis[4] on phase structures (marked in **a**). **d** Distribution of atoms according to their phase stability $\Delta E_{\text{FCC-BCC}}$ for the 1,200K-2M and 1,200K-4M samples, as compared with the 2M and 4M samples. Atomic configurations of the **e** 1,200K-2M sample, and **f** 1,200K-4M sample colored according to phase stability ($\Delta E_{\text{FCC-BCC}}$).

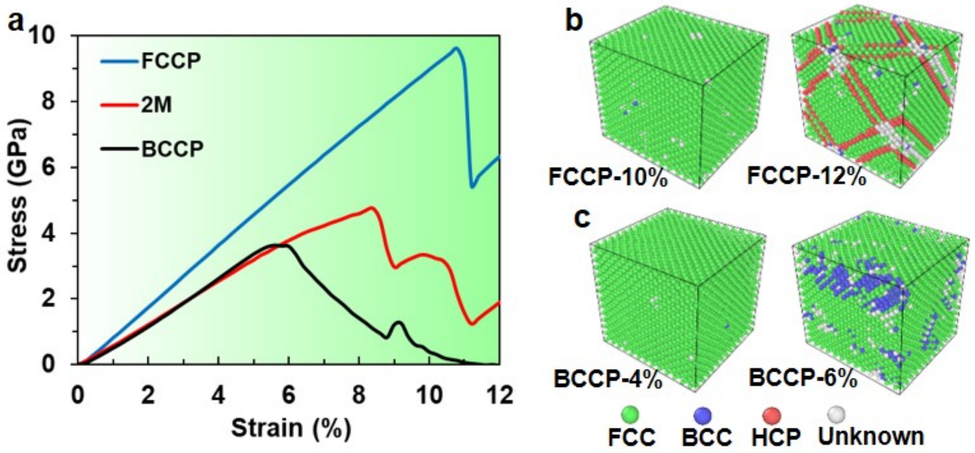

Supplementary Figure 12. Tensile stress-strain curves and atomic configurations of the samples with the same elemental concentrations as FCCP and BCCP clusters in the 2M sample at 300K (labelled as FCCP and BCCP samples). a Stress-strain curves of the FCCP and BCCP samples, along with the results of the 2M sample. Atomic configurations of **b** the FCCP sample and **c** the BCCP sample at different strains colored according to their common neighbor analysis[4] on phase structures.

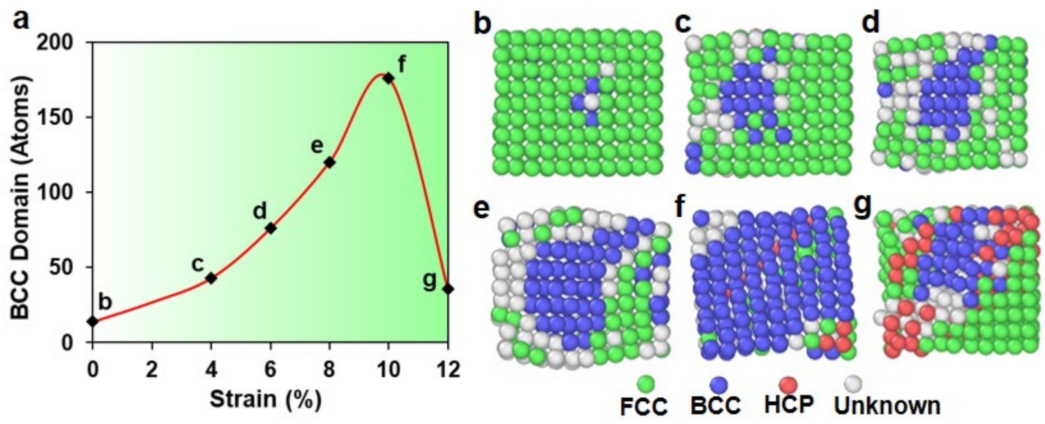

Supplementary Figure 13. Evolution of a BCC domain in the CoCuFeNiPd HEA with 2 × 10^6 iterations at 300 K (the 2M sample) during tension at 300 K. a Variation of the number of BCC atoms in the domain with strain. **b-g** Atomic configurations of the BCC domain at different strains (marked in **a**) according to their common neighbor analysis[4] on phase structures.

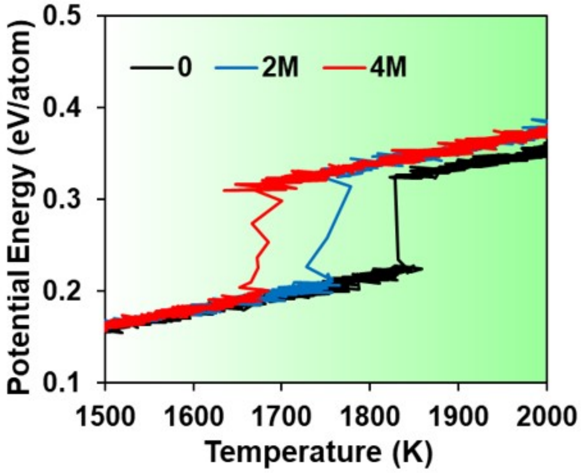

Supplementary Figure 14. Variation of potential energy with temperature for CoCuFeNiPd HEA after 0, 2 × 10^6 (2M) and 4 × 10^6 (4M) iterations.

Supplementary Tables

Supplementary Table 1. Cohesive energies and number of atomic pairs in the CoCuFeNiPd HEA during iterations at 300K. a Calculated cohesive energies of the lowest energy structures of $L1_2$ unit cells (unit: eV/atom) from DFT calculations. Number of atomic pairs in the HEA for **b** the initial configuration and the configurations after **c** 2×10^6 and **d** 4×10^6 iterations.

Pd	-4.339	-3.647	-4.346	-4.193	-3.631
Ni	-4.989	-4.131	-4.950	-4.818	-4.193
Fe	-5.050	-4.090	-4.883	-4.950	-4.346
Cu	-4.181	-3.494	-4.090	-4.131	-3.647
Co	-5.160	-4.181	-5.050	-4.989	-4.339
**a**	Co	Cu	Fe	Ni	Pd

-5.2 –– -3.4

Pd	4238	4269	4240	4205	4168
Ni	4193	4252	4150	4284	4205
Fe	4156	4162	4376	4150	4240
Cu	4201	4200	4162	4252	4269
Co	4296	4201	4156	4193	4238
**b**	Co	Cu	Fe	Ni	Pd

500 –– 13000

Pd	1275	3993	8253	921	6678
Ni	5528	2072	1715	10848	921
Fe	2312	2708	6096	1715	8253
Cu	2069	10242	2708	2072	3993
Co	9900	2069	2312	5528	1275
**c**	Co	Cu	Fe	Ni	Pd

500 –– 13000

Pd	1425	3364	8617	630	7084
Ni	5501	2045	1548	11360	630
Fe	2560	1769	6590	1548	8617
Cu	1520	12386	1769	2045	3364
Co	10078	1520	2560	5501	1425
**d**	Co	Cu	Fe	Ni	Pd

500 –– 13000

Supplementary Table 2. Comparison of cohesive energies of the $L1_2$ unit cell. **a** MD simulations. **b** DFT calculations.

a	Co	Cu	Fe	Ni	Pd
Co_3	-4.39	-4.16	-4.35	-4.41	-4.24
Cu_3	-3.73	-3.54	-3.69	-3.75	-3.63
Fe_3	-4.25	-4.02	-4.19	-4.26	-4.19
Ni_3	-4.43	-4.19	-4.38	-4.45	-4.25
Pd_3	-4.01	-3.82	-4.05	-4.00	-3.91

b	Co	Cu	Fe	Ni	Pd
Co_3	-5.16	-4.63	-5.13	-5.09	-4.72
Cu_3	-3.73	-3.49	-3.66	-3.81	-3.63
Fe_3	-4.97	-4.52	-4.88	-4.94	-4.61
Ni_3	-4.89	-4.45	-4.96	-4.82	-4.49
Pd_3	-3.96	-3.66	-4.08	-3.90	-3.63

Supplementary References

[1] Li, X., Wei, Y., Lu, L., Lu, K. & Gao, H. Dislocation nucleation governed softening and maximum strength in nano-twinned metals. *Nature* **464**, 877–880 (2010).

[2] Zhao, S. et al. Amorphization in extreme deformation of the CrMnFeCoNi high-entropy alloy. *Sci. Adv.* **7**, eabb3108 (2021).

[3] Yin, B., Maresca, F., Curtin, W. A. Vanadium is an optimal element for strengthening in both fcc and bcc high-entropy alloys. *Acta Mater.* **188**, 486–491 (2020).

[4] Stukowski, A. Visualization and analysis of atomistic simulation data with OVITO-the open visualization tool. *Model. Simulat. Mater. Sci. Eng.* **18**, 15012 (2009).

[5] Ding, Q. et al. Tuning element distribution, structure and properties by composition in high-entropy alloys. *Nature* **574**, 223–227 (2019).

[6] Verma, A. et al. High temperature wear in $CoCrFeNiCu_x$ high entropy alloys: The role of Cu. *Scr. Mater.* **161**, 28–31 (2019).

[7] Dąbrowa, J. et al. Demystifying the sluggish diffusion effect in high entropy alloys. *J. Alloys Compd.* **783**, 193–207 (2019).

[8] Munitz, A., Kaufman, M. J., Abbaschian, R. Liquid phase separation in transition element high entropy alloys. Intermetallics 86, 59–72 (2017).

[9] Tong, C.-J. et al. Microstructure characterization of $Al_xCoCrCuFeNi$ high-entropy alloy system with multiprincipal elements. *Metall. Mater. Trans. A* **36**, 881–893 (2005).

[10] Sohn, S. et al. Noble metal high entropy alloys. *Scr. Mater.* **126**, 29–32 (2017).
