# Supplementary material for: Simultaneously enhancing the ultimate strength and ductility of high-entropy alloys via short-range ordering
Source: Nat Commun. 2021 Aug 16;12:4953. doi: 10.1038/s41467-021-25264-5 (PMC8368001; doi:10.1038/s41467-021-25264-5)
Supplement: Supplementary file 3 — Description of Additional Supplementary Files [file 41467_2021_25264_MOESM3_ESM.pdf]

Description of Additional Supplementary Files

File Name: Supplementary Data 1
Description: The cohesive-energy data of FCC and BCC structures.
